# Physico-Chemical Properties and Storage Stability of an Emulsion as a Fat Replacer in Meat Analogs during the Freezing Storage

**DOI:** 10.3390/foods11243977

**Published:** 2022-12-08

**Authors:** Hyeseung Jeong, Haesanna Kim, Jiseon Lee, Yeon-Ji Jo, Mi-Jung Choi, Eun-Young Ko

**Affiliations:** 1 Department of Food Science and Biotechnology of Animal Resources, Konkuk University, Seoul 05029, Republic of Korea; 2 Carbohydrate Bioproduct Research Center, Sejong University, Seoul 05006, Republic of Korea; 3 Department of Food Processing and Distribution, Gangneung-Wonju National University, Gangneung 25457, Republic of Korea

**Keywords:** meat analog, fat replacer, O/W emulsion, freezing storage

## Abstract

This study determined the effects of physicochemical and microbial properties of emulsion as a fat replacer in meat analogs during freezing storage. Meat analogs were prepared with different fat replacers: vegetable oil (O) for control, oil in water emulsion (E), and non-emulsified oil in water emulsion (EC) for emulsion control. After that, meat analogs were stored for 0.5, one, three, and six months at −18 °C and −60 °C. The results showed that the drip loss of all samples was not significantly different (*p* > 0.05). However, the liquid holding capacity of EC and E was significantly higher than that of O (*p* < 0.05). Additionally, the microstructures of meat analogs of E and EC were smaller with denser pore sizes than O. This explains the significantly lower hardness of E and EC compared to O (*p* < 0.05). Overall, E showed superior physiochemical and sensory quality. During the storage, the stability of chemical properties, such as volatile basic nitrogen and thiobarbituric acid reactive substances, showed no significant changes (*p* > 0.05). Moreover, the microbial studies (total viable counts and *Escherichia coli* count) suggested that meat analogs did not deteriorate during the preparation and storage. Thus, this study suggests that emulsion-type fat replacers influence meat analogs’ physicochemical and sensorial properties. However, these properties are not influenced by the storage temperature and duration.

## 1. Introduction

Increasing public health concerns related to environmental and ethical issues and high consumption of saturated and *trans* fatty acids have led to the increased consumption of plant-based and meat-alternative foods [1]. A meat alternative is a food similar in nutritional composition or substance to meat from plant-based sources [2]. Meat alternatives imply analogs and reformulated products, such as patties, sausages, and nuggets, which are classified based on protein sources as plant-based, cell-based, and fermentation-based [3]. The essential nutrients in meat analogs are proteins and fats, which play significant roles in nutritional, physical, and sensory characteristics [4]. Therefore, in meat analogs, various vegetable oils, such as olive, linseed, chia seed, canola, and sunflower oils, are used as fat replacers [5,6]. However, a few researchers have reported that applying vegetable oils to meat products can deteriorate the products, resulting in reduced elasticity, nutritional loss, and shortened storage periods due to oxidation [7].

Food emulsion comprises two immiscible ingredients, such as oil and water, which are classified as oil dispersed in water (O/W), or water dispersed in oil (W/O) [8]. For replacing animal fat, the emulsion can be used as a fat replacer. Studies have suggested that emulsion application in meat products and meat analogs facilitates the production of healthier and low-fat food products [9,10]. Emulsion-type fat replacers indicate nutritional values and functional values by improving the liquid holding capacity (LHC), oxidation stability, and sensory properties of meat products [10,11].

The freezing process can contribute to maintaining the physical and biochemical reactions of food products [12]. However, freezing and thawing can cause irreversible damage, such as color change, texture loss, and drip loss. However, the freezing speed affects the degree of tissue damage [13]. Rapid freezing distributes uniform fine ice crystals and reduces the dislocation of water, which results in a higher WHC [14].

Very few studies have investigated the physicochemical properties and storage stability of meat analogs with emulsion during freezing storage. Additionally, oil in water (O/W) emulsions are predisposed to destabilize during the freezing and thawing of the oil droplets, which crystallize in advance of the water phase [15,16]. Therefore, this study formulated meat analogs with different fat replacers, including vegetable oil (O) for control, oil in water emulsion (E), and non-emulsified oil in water emulsion (EC) for emulsion control, and investigated the influence of physicochemical properties and storage stability of meat analogs during freezing storage for six months. This study will facilitate an understanding of the influence of long-term storage in meat analogs.

## 2. Materials and Methods

### 2.1. Materials

Textured vegetable protein (TVP, SUPRO^®^ MAX 5050 and SUPRO^®^ MAX 5010, DuPont Korea, Seoul, Republic of Korea), binder (GRINDSTED^®^ Meatline 2714, DuPont Korea, Seoul, Republic of Korea), and SPI (ESfood, Gangwon, Republic of Korea) were used to fabricate the meat analogs. Textured vegetable protein (TVP) contains SPI (55–60%), wheat gluten (40–45%), and wheat starch. The binder was a mixture of egg-white powder, glucose, soy protein, locust bean gum, carrageenan, and guar gum. Canola oil (Sajohaepyo Corporation, Seoul, Republic of Korea), Tween 80^®^ (Daejung Chemicals & Materials, Gyeonggi, Republic of Korea) was used to fabricate the emulsion. All other chemical reagents were of analytical grade and were used without further purification.

### 2.2. Preparation of Fat Replacer

Fat replacers applied to meat analogs are shown in Table 1. The canola oil (O) was used as a control. The emulsion control (EC) was the non-homogenized mixture of canola oil, distilled water in the ratios of 4:6 (oil phase: water phase; *v*/*v*), and Tween 80^®^. Oil in water emulsion (E) was prepared using canola oil and distilled water in the ratio of 4:6 (oil phase: water phase; *v*/*v*) with Tween 80^®^ as an emulsifier. The mixture was homogenized at 12,000× *g* for 3 min using a high-speed homogenizer (T25 digital ULTRA-TURRAX^®^, IKA, Staufen, Germany).

### 2.3. Preparation of Meat Analogs

Meat analogs were prepared by following the method with slight modifications [17]. TVPs were immersed in water (10 times in volume) for hydration for 2 h. After that, TVPs were dehydrated using a centrifugal dehydrator (ws-6600, Hanil Electric, Seoul, Republic of Korea) at 1200× *g* for 5 min. The mixing ratios of meat analogs are shown in Table 1. Mixtures were blended for 1 min using a hand blender (550 W, Multiquick 3 Vario, Braun, Kronberg im Taunus, Germany) and were molded into the cylindrical mold (60 mm diameter and 15 mm height). Furthermore, molded mixtures were cooked in an oven (M4207, Simfer, Istanbul, Turkey) at 180 °C for 14 min and cooled to room temperature. After cooling, samples were packed into vacuum-sealing packaging (Solis vacuum rolls, Solis, Glattbrugg-Zurich, Switzerland) made of foodsafe BPA-free plastic (three-layered film with a 110 μm). One cylindrical mold was packed and stored in refrigerators (R–F875HBSW, LG Electronics, Seoul, Republic of Korea) set at −18 °C and −60 °C for 0.5 (14 days), 1, 3, and 6 months. Stored samples were thawed in refrigerators (R–F875HBSW) set to 4 °C for 12 h before analysis. Meat analog production was carried out three times.

### 2.4. Visible Appearance

Images of the external appearance of the emulsion gels were acquired with a digital camera (α350, Sony, Tokyo, Japan) and the characteristics were observed.

### 2.5. Color Measurements

A colorimeter (CR-400, Konica Minolta Sensing, Inc., Tokyo, Japan) was used under constant illumination (light source simulating the relative spectral irradiance of D65 CIE standard illuminant). Determination was achieved by using the Commission Internationale de l′Eclairage (CIE) system; colors are described using the Hunter (L* (lightness), a* (redness), and b* (yellowness)) values. Five points on the surfaces of meat analogs were evaluated (*n* = 15).

### 2.6. Drip Loss

Drip loss was determined by the weight difference and calculated from the weights of the samples in grams before (W_1_) and after (W_2_) removed exudates.

Drop loss (%) = {(W_1_ − W_2_)/W_1_} × 100 (1)

### 2.7. Water Holding Capacity

The WHC of the emulsion gel was determined according to the method of Jo et al. [18], with slight modification. The samples were weighed (1 g), placed in a centrifugal filter (Vivaspin^®^ 20, Sartorius Stedim Lab Ltd., Stonehouse, UK), and then centrifuged (LaboGene 1736R, GYROGEN, Daejeon, Republic of Korea) at 3000× *g* for 10 min at 20 °C. The WHC was calculated from the weights of the samples in grams before (W_1_) and after (W_2_) centrifugation and that of the empty filters (W_0_).

WHC (%) = {(W_2_ − W_0_)/W_1_} × 100 (2)

### 2.8. Moisture Content

Moisture content was determined by measuring the weight loss of the sample after drying [19]. Homogenized samples (1 g) were weighed and dried in the oven at 105 °C until constant weight (about 5 h). The moisture content was calculated from the weight of samples before (W_1_) and after (W_2_) drying.

Moisture content (%) = {(W_1_ − W_2_)/W_1_} × 100 (3)

### 2.9. Texture Profile Analysis

Texture profile analysis (TPA) was conducted using a texture analyzer (CT3, Brookfield Engineering Labs Inc., Middleboro, MA, USA). Block-shaped samples (width, length, and height: 1.5 × 1.5 × 0.5 cm^3^) were prepared to measure the hardness, cohesiveness, springiness, and chewiness of the samples. TPA of the meat analogs were conducted at room temperature (20 °C) after thawing with a deformation rate of compression of 30% using a TA4/1000 cylindrical probe with a trigger load and test speeds of 5 g and 2.0 mm/s, respectively. Determinations were conducted in eight replicate analyses.

### 2.10. Scanning Electron Microscopy

The microstructure of the emulsion gel was observed using scanning electron microscopy, SEM (TM4000Plus, Hitachi, Tokyo, Japan). Emulsion gels were frozen in liquid nitrogen and then dried in a freeze-dryer (FDCF-12012, Operon Gyeonggi, Republic of Korea) under 5 Pa at −80 °C for 48 h. To remove residual oils, the dried gels were soaked in ethyl ether for 4 h and dried in a dry oven (ThermoStableTM OF-105, DAIHAN^®^, Gangwon, Republic of Korea) for 4 h at 50 °C [20]. The fractured dried samples were observed with a backscattered electron (BSE) detector at a voltage of 15 kV. The micrograph of the samples was taken at 500× magnification.

### 2.11. Volatile Basic Nitrogen

Volatile basic nitrogen (VBN) was determined according to the Conway method of micro-diffusion with slight modification [21]. The sample (4 g) was homogenized with 16 mL of distilled water and then left for 30 min to elute. The homogenate (20 mL) was filtered using the Whatman No. 1 filter paper (GE Healthcare Life Science, Sheffield, UK). A filtered sample (1 mL) was placed in the outer section of the Conway dish with the mixture of 0.01 N H_3_BO_3_ (1 mL) and Conway solution (100 μL) and the mixture of 0.066% methyl red and 0.066% bromocresol green in aqueous ethanol was dropped into the inner section. Additionally, 50% K_2_CO_3_ (1 mL) was added to the outer section of the dish. After that, the Conway dish was incubated at 37 °C for 2 h and titrated using 0.02 N H_2_SO_4_ until the Conway reagent changed to a red color. The VBN values were calculated from (A) the titration volume of 0.02 N H_2_SO_4_ (mL), (B) the titration volume of the blank (mL), (f) the factor of H_2_SO_4_, the weight of the sample (g), and (C) the dilution amount.

VBN (mg/100 g) = {14.007 × (A − B) × f × 100 × C}/S(4)

### 2.12. Thiobarbituric Acid Reactive Substances

Secondary lipid oxidation was determined from the 2-thiobarbituric acid reactive substances (TBARs) value obtained based on a slight modification of the method of Lee et al. [21]. The sample (4 g) was homogenized with 16 mL of distilled water, and then left for 30 min to elute. The homogenate (20 mL) was filtered, and 0.5 mL of the sample was mixed with 4.5 mL of TBA solution (0.25 N hydrochloric acid, 15 % trichloroacetic acid, and 0.375% TBA regent) to analyze the TBARs. Thereafter, the sample was heated at 95 °C for 15 min in a water bath (Shaking Water Bath MaXturdy 45, DAIHAN^®^, Gangwon, Republic of Korea). The heated mixture was cooled at room temperature for 30 min, followed by centrifugation at 3000× *g* for 10 min at 25 °C. The absorbance of the supernatant at 532 nm was read with a spectrophotometer (Multiskan™ GO UV/VIS, Thermo Fisher, Waltham, MA, USA).

### 2.13. Microbial Analysis

Total viable counts (TVC) and *Escherichia coli* counts were conducted according to the method of the Association of Official Analytical Chemists (AOAC) International [19]. Sample (1 g) was homogenized with sterilized 0.85% NaCl solution (9 mL) for 3 min using a slap-type homogenizer (WS-400, Shanghai Zhisun Equipment, Shanghai, China). The supernatant was diluted, by serial dilution and the diluted solution was spread on an agar plate and 3 M Petrifilm™ *E. coli* Count Plates (3 M Health Care, St. Paul, MN, USA). The plates were incubated at 37 °C for 24–48 h. The colonies were counted and expressed as a log of forming units per gram of sample (log CFU/g).

### 2.14. Sensory Evaluation

Ten experienced panelists recruited from the Department of Food Science and Biotechnology of Konkuk University performed the sensory evaluation. Sensory evaluation was conducted individually using the seven-point scoring test by evaluating the parameter intensity and sensory preferences. The samples were cut into cubes (1.5 × 1.5 × 0.5 cm^3^) and permitted to rest for 30 min at 20 °C. Then, the samples were randomly assigned to the panelists. Sensory evaluations included color, hardness, chewiness, tenderness, juiciness, and overall acceptability. Sensory attributes were graded on preference (7: very good, 1: very unacceptable). The Institutional Review Board (IRB) approved the consent procedure for sensory evaluation (nos. 7001355-202111-HR-489).

### 2.15. Statistical Analysis

All experiments were repeated at least thrice (n ≥ 3), and the results were expressed as mean ± standard deviation. Statistical analysis of data was conducted using IBM SPSS statistics version 24.0 (SPSS, INC., Chicago, IL, USA). Independent *t*-tests, one-way ANOVA, and Duncan’s multiple range tests were performed to confirm statistically significant differences (*p* < 0.05).

## 3. Results and Discussion

### 3.1. Visible Appearance and Color Measurements

**Figure 1:**
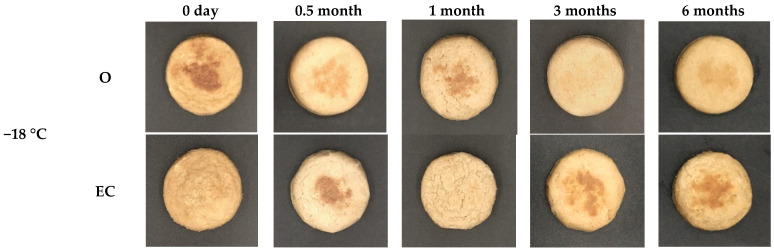

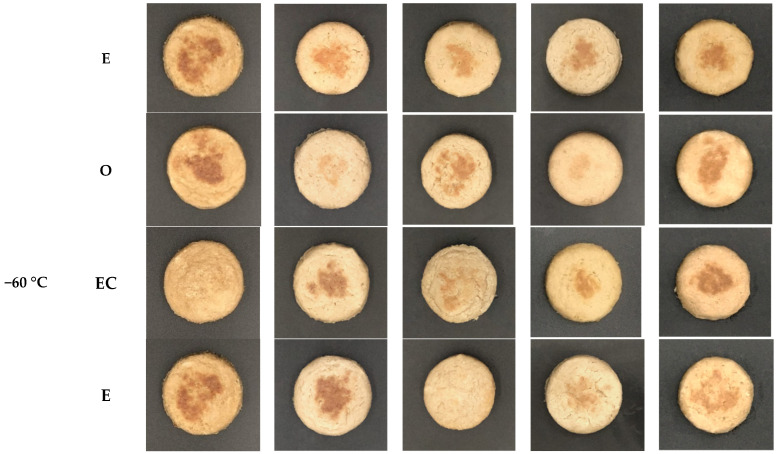
Appearance of meat analogs with different fat replacers (O, EC and E) during the freezing storage (−18, and −60 °C) for six months.

The meat’s color determines the visual preference for the quality during storage [22]. The color and appearance of meat analogs change with different fat replacers and storage temperatures, as shown in Figure 1 and Table 2. The colors of meat analogs with different fat replacers did not show a significant difference (*p* > 0.05). During storage, the lightness of meat analogs was significantly higher during all periods except on 0 d (*p* < 0.05). This was also reflected in the visible appearances (Figure 1). The surface of all meat analogs shrunk and brightened during the next one-month period. Additionally, the yellowness of EC was significantly lower during all periods compared to O and E (*p* < 0.05). The results were similar to the studies of Li et al. [23] and Wang et al. [24] who reported that the lightness of meat patties increased after the freezing and thawing process. This might be related to the ice crystals formed on the samples. As the ice crystals are formed from the extracellular to the intercellular, a large amount of electrolyte is concentrated in the extracellular part. Consequently, intracellular water is released into the extracellular part through osmotic pressure [25]. Additionally, the differences in the color and visible appearance of meat analogs were not affected by the freezing temperature difference (−18 °C and −60 °C).

### 3.2. Drip Loss

**Figure 2:**
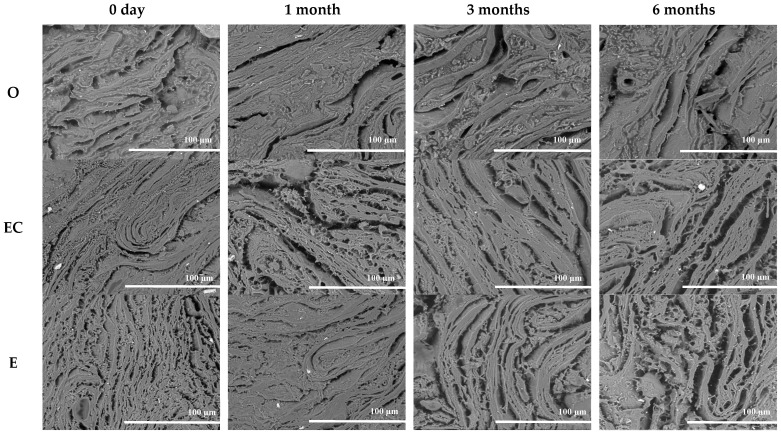
Microstructure of meat analogs with different fat replacers (O, EC, and E) during the freezing storage (−18 °C) for six months (500).

**Figure 3:**
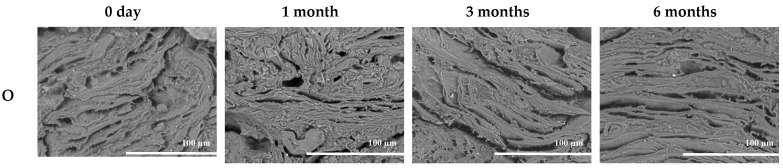

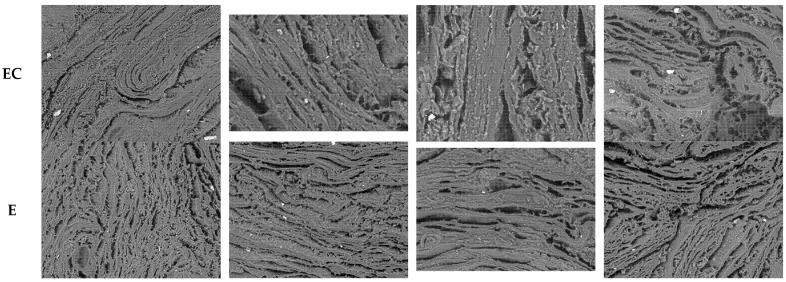
Microstructure of meat analogs with different fat replacers (O, EC, and E) during the freezing storage (−60 °C) for six months (500× magnification).

The drip loss of meat analogs with different fat replacers after freezing and thawing is presented in Table 3. The drip loss of meat analogs with different fat replacers did not show a significant difference (*p* > 0.05). Additionally, there was no significant difference during the storage periods (*p* > 0.05). However, the meat analogs showed a numerical increase in drip loss after 14 days during the freezing storage at −18 °C and −60 °C. The drip loss of the meat analogs stored at −18 °C was numerically higher than at −60 °C. A few studies have demonstrated that drip loss depends on the freezing rate; increased freezing rate causes decreased drip loss [26,27]. This phenomenon is caused by ice recrystallization that would be more easily promoted at a higher temperature rate [28]. This can be explained by the microstructure images, which show a denser and more compact matrix at −60 °C (Figure 2 and Figure 3). Therefore, the results can be explained by the fact that the freezing storage at −60 °C was more stable compared to that at −18 °C.

### 3.3. Liquid Holding Capacity

The LHC of a meat product is a significant indicator of food texture, quality, and mouthfeel [29]. The LHC of meat analogs with different fat replacers after the freezing and thawing is shown in Table 4. Among different fat replacers, O had significantly low LHC during the storage periods of 0.5, one, and three months compared to EC and E at −18 °C and −60 °C. This result explained that emulsion and emulsion control had higher LHC during long-term freezing storage by preventing water loss. Emulsifier EC and E help trap water and oil in the meat analog matrix. Wi et al. [17] and Jimenez-Colmenero et al. [30] explained that the surfactant assists in the dispersion of oil and water into the matrix, which can cause decreased syneresis in the mixture. Additionally, the microstructure of O showed a bigger pore size and cracks than EC and E, which became bigger during storage (Figure 2 and Figure 3). This implies that EC and E generated less damage in the matrix by ice recrystallization during freezing storage at −18 °C. However, subsequent studies are required on the effect of freezing the emulsion in the matrix.

### 3.4. Moisture Contents

Moisture contents play a significant role in food quality because of the chemical reaction from moisture, such as lipid oxidation and texture [31]. Table 5 presents the moisture contents of meat analogs with different fat replacers during long-term freezing storage. As the addition of water was higher in E and EC at the formulation, the moisture contents of E and EC were significantly higher than that of O (*p* < 0.05). Additionally, there was no significant difference during the storage in all treatments, which was also stable without any difference in moisture contents (*p* > 0.05).

### 3.5. Texture Profile Analysis

TPA is used to estimate imitation foods by verifying whether the texture properties emulate food perception by the consumers [32]. The hardness of meat analogs was significantly high in O (*p* < 0.05). The addition of emulsion could impart tenderness to the meat analogs compared to non-emulsified oil and water; E. Cohesiveness, chewiness, and springiness of meat analogs also showed similar patterns regarding hardness (Table 6). These results might be due to the moisture contents of E and EC. Lin et al. [33] reported that higher moisture contents decrease texture properties (hardness, cohesiveness, chewiness, and gumminess) of meat analogs. Moreover, Lee et al. [34] reported a similar result that the pork patty formulated with nanoemulsion showed lower hardness. During the periods, the hardness of meat analogs with O and EC decreased compared to the control samples (0 days) without E. This result might be caused by the fact that emulsification can trap water in the matrix [17,30].

Additionally, the increased hardness of meat analogs might be due to the loss of water from the melted ice crystal from the matrix after the thawing during storage [35,36]. Moreover, the samples stored at −18 °C showed lower hardness compared to those stored at −60 °C. The size and distribution of ice crystals affected the freezing temperature, which can be indicated by the matrix microstructure in Figure 2 and Figure 3. Moreover, these results can be explained by the results of drip loss and the WHC that have similar increasing patterns during different study periods.

### 3.6. Microstructure

The microstructures of meat analogs with different fat replacers and freezing temperature conditions (−18 °C and −60 °C) are shown in Figure 2 and Figure 3. The samples with O seemed to have a thick and assembled matrix. However, the matrix and pores of EC and E appeared more neatly arranged, compact, and denser. Owing to moisture content differences, the water of EC and E was more considerably embedded into the matrix by homogenization. Wi et al. [17] and Jimenez-Colmenero et al. [30] reported that the microstructure of meat analogs and frankfurters with emulsion indicated that the emulsifier of emulsion might be assisted by the dispersion of oil and water into the matrix. Additionally, the number of smaller pores affected the lower strength [17,33].

During the storage periods, the pore size was more prominent, and the matrix layers were thicker than the non-freezing sample (0 days), as the decreased holding capacity was caused by ice-recrystallization. The formation of large ice crystals and irregular distribution can irreversibly break the structure of the matrix [37,38]. These results were consistent with hardness, cohesiveness, and chewiness results. The more extended periods showed the higher hardness of the meat analogs.

### 3.7. Volatile Basic Nitrogen

VBN is used as an indicator of freshness on protein degradation during the long-term freezing storage of meat analogs [39]. The results of VBN are presented in Table 7. Different fat replacers did not show significant differences and showed slight changes in VBN during all periods. Therefore, six months of freezing storage did not lead to protein degradation either by enzymatic mechanisms or microorganisms, as evidenced by no microbial growth [40].

### 3.8. Thiobarbituric Acid Reactive Substances

TBARs present the formation of secondary oxidation products such as malondialdehyde (MDA) generated by lipolytic enzymes, microbial metabolism, and oxidation [41]. TBARs values of meat analogs with different fat replacers (O, EC, and E) during the freezing storages are presented in Table 8. Significant differences between fat replacers were shown after one-month storage, when EC and E were significantly higher than O (*p* < 0.05). Similarly, after three months, EC was significantly higher than E and O (*p* < 0.05). Jo et al. [42] explained that the partial replacement of pork back fat with the fish oil emulsion showed decreased TBAR values compared to replacement with fish oil during the storage.

However, the TBARs of all meat analogs did not exceed 1.0 mg/kg. Meat with TBAR values below 1.0 mg/kg is considered fresh and fit for consumption [43]. Therefore, these results confirm that all meat analogs under freezing conditions were stable for six months regardless of the kinds of fat replacer used.

### 3.9. Microbial Analysis

The hygiene of patty products is a concern for public health because patty production involves several steps [44]. The TVC and *E. coli* count of meat analogs with different fat replacers are shown in Table 9. The TVC of EC was significantly higher than that of O and E in one and six months at −18 °C (*p* < 0.05). This can be explained by the fact that free water in the non-emulsified emulsion enhanced aw and the aw leads to the deterioration of microbial growth [45].

During storage, the TVC levels of meat analogs changed slightly but significantly regardless of the initial value (*p* < 0.05). This was probably because microorganism activities were inhibited by the frozen storage temperature (−18 °C and −60 °C) [46]. These results indicate that fat replacement with lots of water, such as emulsion, provided microbial safety and stability during long-term freezing storage. Additionally, according to the storage temperature difference, there were no significant differences. However, the storage temperature of −60 °C was constant during initial storage from 0.5 to three months.

The *E. coli* count of meat analogs was not detected during all storage periods, which implies that meat analogs were not spoiled during the preparation process.

### 3.10. Sensory Evaluation

**Figure 4:**
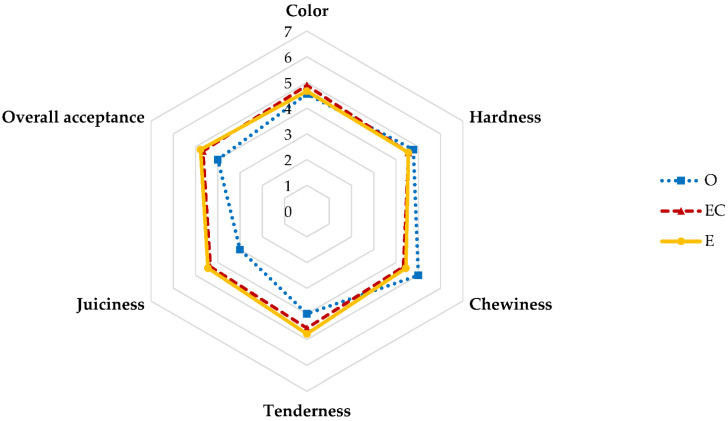
Sensory evaluation of meat analogs with different fat replacers (O, EC, and E) during the freezing storage (−18 °C) for six months. O: oil, EC: non-emulsified emulsion control; and E: emulsion.

The sensory evaluation was conducted to analyze the difference in the preference of meat analogs after the six-month freezing storage. The results of the sensory evaluation are shown in Figure 4. The preferences of hardness and chewiness of O were higher than EC and E. However, the tenderness, juiciness, and overall acceptance of E were highest compared to O and EC. Lee et al. [34] and Kim et al. [47] also reported that the nanoemulsion applied pork patty influenced the preference score of juiciness and tenderness in sensory evaluation. The tenderness and juiciness E probably affected overall acceptance [48].

## 4. Conclusions

The present investigation aimed to determine the influence of different fat replacers during long-term freezing storage. The meat analogs were formulated with vegetable oil (O) for control, oil in water emulsion (E), and non-emulsified oil in water emulsion (EC) for emulsion control and stored at −18 °C and −60 °C for six months. Different fat replacers showed a significant influence on appearance and color, liquid holding capacity (LHC), hardness, moisture content, microstructure, tenderness, juiciness, and overall acceptance of meat analogs. Meanwhile, drip loss, volatile basic nitrogen (VBN), and thiobarbituric acid reactive substances (TBARs) were not influenced by different fat replacers. Overall, E showed superior physiochemical and sensory quality.

Interestingly, there was no significant difference between the storage temperature (−18 °C and −60 °C) and physicochemical and microbial properties. The results of this study present the possibility of utilizing emulsion as a fat replacer in meat analogs.

## Figures and Tables

**Table 1:** Final component of the meat analog and fat replacer.

Treatments *	Meat Analog Ingredients (%)			Fat Replacer Ingredients (%)		
	TVP	SPI	Binder	Distilled Water	Canola Oil	Tween^®^ 80
O	74.67	3.00	2.33	-	20.00	-
EC	74.67	3.00	2.33	11.88	8.00	0.12
E	74.67	3.00	2.33	11.88	8.00	0.12

* Treatments: meat analogs with different fat replacers, O (oil); EC (non-emulsified emulsion control); E (emulsion).

**Table 2:** Color of meat analogs with different fat replacers (O, EC, and E) during the freezing storage (−18 °C, −60 °C) for six months.

Storage Temperature (°C)	Color	Treatments *	Storage (Months)				
			0	0.5	1	3	6
−18	Lightness (L*)	O	48.47 ± 1.23^D^	63.58 ± 4.62^aA^	63.86 ± 1.05^A^	58.72 ± 0.86^aB^	56.11 ± 1.52^C^
		EC	48.74 ± 1.28^C^	56.82 ± 1.84^bAB^	53.33 ± 2.20^B^	58.10 ± 0.53^abA^	56.34 ± 1.85^AB^
		E	48.62 ± 2.02^D^	65.72 ± 3.48^aA^	64.49 ± 1.83^A^	57.38 ± 1.54^bB^	55.44 ± 0.95^C^
	Redness (a*)	O	5.50 ± 0.40^aB^	5.82 ± 0.12^aB^	7.73 ± 0.79^aA^	5.12 ± 1.97^B^	3.89 ± 0.14^abC^
		EC	5.19 ± 0.33^bA^	3.82 ± 0.49^bB^	5.24 ± 1.65^bA^	5.63 ± 2.12^A^	3.82 ± 0.22^bB^
		E	5.56 ± 0.12^aB^	5.82 ± 0.08^aB^	7.68 ± 0.46^aA^	4.56 ± 1.28^C^	4.08 ± 0.24^aC^
	Yellowness (b*)	O	17.02 ± 0.36^BC^	17.71 ± 0.18^aA^	17.64 ± 1.42^AB^	16.73 ± 0.73^C^	17.32 ± 0.10^aAB^
		EC	17.52 ± 0.87^A^	16.33 ± 0.69^cB^	16.75 ± 0.74^B^	16.41 ± 0.71^B^	16.61 ± 0.45^bB^
		E	17.40 ± 0.92^AB^	17.74 ± 0.15^bAB^	17.88 ± 1.32^A^	17.08 ± 0.97^B^	17.16 ± 0.27^aAB^
−60	Lightness (L*)	O	48.47 ± 1.23^C^	58.98 ± 3.61^bA^	56.58 ± 2.83^B^	56.16 ± 2.40^B^	56.72 ± 2.57^aB^
		EC	48.74 ± 1.28^D^	56.46 ± 2.27^bC^	58.79 ± 0.37^A^	55.99 ± 0.74^B^	53.31 ± 1.93^bC^
		E	48.62 ± 2.02^D^	64.04 ± 4.69^aA^	57.09 ± 2.84^B^	55.26 ± 3.27^BC^	54.26 ± 0.60^bC^
	Redness (a*)	O	5.50 ± 0.40^aB^	5.92 ± 0.15^aA^	3.49 ± 0.35^E^	4.48 ± 0.86^aC^	4.01 ± 0.26^bD^
		EC	5.19 ± 0.33^bA^	3.61 ± 0.45^bC^	3.60 ± 0.16^C^	3.50 ± 0.13^bC^	4.59 ± 0.71^aB^
		E	5.56 ± 0.12^aA^	5.84 ± 0.12^aA^	3.24 ± 0.37^C^	4.48 ± 0.84^aB^	4.66 ± 0.24^aB^
	Yellowness (b*)	O	17.02 ± 0.36^B^	17.84 ± 0.24^aA^	15.98 ± 0.66^C^	16.52 ± 1.05^aB^	17.06 ± 0.63^bB^
		EC	17.52 ± 0.87^A^	16.28 ± 1.20^bB^	16.12 ± 0.48^B^	15.54 ± 0.35^bB^	17.61 ± 0.69^abA^
		E	17.40 ± 0.92^AB^	17.77 ± 0.18^aAB^	15.74 ± 0.85^C^	17.13 ± 1.09^aB^	17.83 ± 0.60^A^

^a–c^ Means within a column with different letters indicate significant differences (*p* < 0.05). ^A–E^ Means within a row with different letters indicate significant differences (*p* < 0.05). * Treatments: meat analogs with different fat replacers, O (oil); EC (non-emulsified emulsion control); E (emulsion).

**Table 3:** Drip loss (%) of meat analogs with different fat replacers (O, EC, and E) during the freezing storage (−18 °C, −60 °C) for six months.

Storage Temperature (°C)	Treatments *	Storage (Months)				
		0	0.5	1	3	6
−18	O	-	0.52 ± 0.19	0.52 ± 0.17	0.52 ± 0.11	0.59 ± 0.04
	EC	-	0.42 ± 0.27^B^	0.78 ± 0.11^A^	0.60 ± 0.07^AB^	0.58 ± 0.08^AB^
	E	-	0.53 ± 0.29	0.62 ± 0.26	0.68 ± 0.21	0.51 ± 0.11
−60	O	-	0.48 ± 0.05	0.40 ± 0.09	0.33 ± 0.19	0.47 ± 0.22
	EC	-	0.45 ± 0.24	0.52 ± 0.21	0.43 ± 0.13	0.54 ± 0.27
	E	-	0.37 ± 0.20	0.34 ± 0.05	0.55 ± 0.2	0.66 ± 0.17

^A,B^ Means within a row with different letters indicate significant differences (*p* < 0.05). * Treatments: meat analogs with different fat replacers, O (oil); EC (non-emulsified emulsion control); E (emulsion).

**Table 4:** Liquid holding capacity (%) of meat analogs with different fat replacers (O, EC, and E) during the freezing storage (−18 °C, −60 °C) for six months.

Storage Temperature (°C)	Treatments *	Storage (Months)				
		0	0.5	1	3	6
−18	O	98.17 ± 0.89^A^	97.10 ± 0.14^bAB^	97.02 ± 0.38^bAB^	97.15 ± 0.39^AB^	96.25 ± 1.35^B^
	EC	98.16 ± 0.07^AB^	98.00 ± 0.29^aAB^	98.28 ± 0.29^aA^	97.79 ± 0.29^B^	96.51 ± 0.29^C^
	E	97.95 ± 0.36^A^	97.86 ± 0.19^aAB^	97.35 ± 0.23^bB^	97.56 ± 0.54^AB^	96.09 ± 0.35^C^
−60	O	98.17 ± 0.89^A^	97.23 ± 0.17^bB^	96.92 ± 0.35^bB^	97.06 ± 0.21^bB^	95.67 ± 0.27^bC^
	EC	98.16 ± 0.07^AB^	98.19 ± 0.31^aAB^	98.45 ± 0.17^aA^	97.88 ± 0.15^aB^	96.95 ± 0.22^aC^
	E	97.95 ± 0.36^AB^	98.26 ± 0.0^aA^	97.51 ± 0.60^bB^	97.42 ± 0.38^bB^	96.10 ± 0.30^bC^

^a,b^ Means within a column with different letters indicate significant differences (*p* < 0.05). ^A–C^ Means within a row with different letters indicate significant differences (*p* < 0.05). * Treatments: meat analogs with different fat replacers, O (oil); EC (non-emulsified emulsion control); E (emulsion).

**Table 5:** Moisture contents (%) of meat analogs with different fat replacers (O, EC, and E) during the freezing storage (−18 °C, −60 °C) for six months.

Storage Temperature (°C)	Treatments *	Storage (Months)				
		0	0.5	1	3	6
−18	O	48.98 ± 0.69^bA^	48.40 ± 0.22^bA^	46.74 ± 0.26^cB^	48.56 ± 0.30^bA^	48.58 ± 1.04^bA^
	EC	60.75 ± 1.52^a^	61.14 ± 0.86^a^	61.10 ± 0.93^a^	61.87 ± 0.53^a^	62.12 ± 0.14^a^
	E	60.22 ± 0.25^aAB^	60.79 ± 1.49^aAB^	58.80 ± 1.29^bB^	59.83 ± 3.31^aAB^	62.28 ± 0.36^aA^
−60	O	48.98 ± 0.69^bB^	47.84 ± 0.48^cCD^	47.52 ± 0.34^cD^	48.55 ± 0.79^bBC^	49.97 ± 0.12^bA^
	EC	60.75 ± 1.52^aA^	58.98 ± 0.50^aB^	59.65 ± 0.64^aAB^	60.90 ± 0.18^aA^	60.58 ± 0.90^aA^
	E	60.22 ± 0.25^aB^	57.23 ± 1.09^bC^	58.06 ± 0.35^bC^	61.01 ± 0.58^aAB^	61.35 ± 0.48^aA^

^a–c^ Means within a column with different letters indicate significant differences (*p* < 0.05). ^A–D^ Means within a row with different letters indicate significant differences (*p* < 0.05). * Treatments: meat analogs with different fat replacers, O (oil); EC (non-emulsified emulsion control); E (emulsion).

**Table 6:** Texture profile analysis of meat analogs with different fat replacers (O, EC, and E) during the freezing storage (−18 °C, −60 °C) for six months.

Storage Temperature (°C)	TPA	Treatments *	Storage (Months)				
			0	0.5	1	3	6
−18	Hardness (N)	O	60.85 ± 6.99^aB^	62.53 ± 7.51^aB^	61.19 ± 4.18^aB^	65.27 ± 5.88^aB^	75.31 ± 5.21^aA^
		EC	44.65 ± 4.01^bD^	56.54 ± 3.77^aB^	52.26 ± 4.17^bBC^	62.78 ± 7.83^bA^	48.89 ± 4.64^bCD^
		E	37.67 ± 4.27^c^	36.64 ± 5.54^b^	35.64 ± 7.42^c^	39.20 ± 5.66^b^	44.07 ± 13.1^b^
	Cohesiveness	O	0.71 ± 0.03^aA^	0.58 ± 0.02^aBC^	0.61 ± 0.06^aB^	0.54 ± 0.04^aD^	0.54 ± 0.04^aCD^
		EC	0.59 ± 0.03^bA^	0.45 ± 0.03^cB^	0.46 ± 0.02^bB^	0.44 ± 0.02^bB^	0.45 ± 0.03^bB^
		E	0.51 ± 0.04^cA^	0.50 ± 0.01^bAB^	0.48 ± 0.02^bBC^	0.47 ± 0.01^bC^	0.47 ± 0.02^bBC^
	Springiness (mm)	O	5.49 ± 0.05^aA^	5.26 ± 0.04^aC^	5.28 ± 0.12^aBC^	5.33 ± 0.06^aBC^	5.35 ± 0.09^aB^
		EC	5.05 ± 0.11^bA^	4.57 ± 0.05^bC^	4.48 ± 0.11^abC^	4.51 ± 0.14^bC^	4.71 ± 0.09^bB^
		E	4.62 ± 0.14^cA^	4.46 ± 0.07^cBC^	3.87 ± 1.38^bC^	4.52 ± 0.15^bAB^	4.50 ± 0.08^cABC^
	Chewiness (mJ)	O	236.48 ± 30.3^aA^	201.14 ± 14.9^B^	175.28 ± 75.9^aB^	196.00 ± 20.1^aB^	218.34 ± 14.5^aAB^
		EC	132.56 ± 13.3^bA^	117.63 ± 3.99^ABC^	107.58 ± 9.45^bC^	125.36 ± 19.8^bAB^	109.90 ± 19.0^bBC^
		E	88.45 ± 16.6^c^	80.88 ± 14.8	73.44 ± 12.6^b^	77.48 ± 16.9^c^	87.43 ± 28.4^b^
−60	Hardness (N)	O	60.85 ± 6.99^a^	61.01 ± 9.25^a^	60.05 ± 5.67^a^	67.07 ± 5.73^a^	61.71 ± 10.2^a^
		EC	44.65 ± 4.01^b^	46.24 ± 6.99^b^	46.86 ± 4.77^b^	51.28 ± 11.3^b^	49.19 ± 3.54^b^
		E	37.67 ± 4.27^cAB^	35.53 ± 5.19^cAB^	32.71 ± 4.11^cB^	33.46 ± 4.98^cB^	40.60 ± 7.33^bA^
	Cohesiveness	O	0.71 ± 0.03^aA^	0.57 ± 0.02^aB^	0.59 ± 0.09^aB^	0.57 ± 0.03^aB^	0.55 ± 0.06^B^
		EC	0.59 ± 0.03^bA^	0.44 ± 0.02^cB^	0.45 ± 0.03^bB^	0.45 ± 0.04^aB^	0.43 ± 0.02^bB^
		E	0.51 ± 0.04^aB^	0.49 ± 0.02^bB^	0.61 ± 0.02^aA^	0.46 ± 0.02^aC^	0.45 ± 0.02^bC^
	Springiness (mm)	O	5.49 ± 0.05	5.27 ± 0.06^a^	5.32 ± 0.07^a^	5.32 ± 0.06^a^	5.37 ± 0.04^a^
		EC	4.50 ± 1.59^A^	4.36 ± 0.14^bB^	5.06 ± 0.15^aB^	4.26 ± 0.15^bB^	4.29 ± 0.08^bB^
		E	4.62 ± 0.14	4.13 ± 0.10^c^	4.33 ± 0.07^b^	4.16 ± 0.18^b^	4.31 ± 0.16^b^
	Chewiness (mJ)	O	236.48 ± 30.3^aA^	189.54 ± 24.2^aB^	197.30 ± 24.90^aB^	203.89 ± 16.9^aB^	178.65 ± 15.95^aB^
		EC	132.56 ± 13.3^bA^	95.53 ± 12.5^bB^	91.90 ± 12.78^bB^	98.39 ± 24.0^bB^	91.80 ± 10.50^bB^
		E	88.45 ± 16.6^cAB^	72.94 ± 12.5^cCD^	94.64 ± 13.73^bA^	60.09 ± 6.92^cD^	79.30 ± 15.72^BC^

^a–c^ Means within a column with different letters are significantly different (*p* < 0.05). ^A–D^ Means within a row with different letters are significantly different (*p* < 0.05). * Treatments: meat analogs with different fat replacers, O (oil); EC (non-emulsified emulsion control); E (emulsion).

**Table 7:** VBN (mg/%) of meat analogs with different fat replacers (O, EC, and E) during the freezing storage (−18 °C, −60 °C) for six months.

VBN (mg/%)	**Storage** **Temperature (°C)**	Treatments *	**Storage (Months)**				
			**0**	**0.5**	**1**	**3**	**6**
	−18	O	0.00 ± 0.00	0.00 ± 0.00	0.06 ± 0.14	0.12 ± 0.00	0.00 ± 0.00
		EC	0.06 ± 0.14	0.06 ± 0.14	0.00 ± 0.00	0.12 ± 0.00	0.00 ± 0.00
		E	0.06 ± 0.14	0.06 ± 0.14	0.00 ± 0.00	0.12 ± 0.00	0.00 ± 0.00
	−60	O	-	0.00 ± 0.00	0.00 ± 0.00	0.12 ± 0.00	0.00 ± 0.00
		EC	-	0.00 ± 0.14	0.06 ± 0.14	0.12 ± 0.00	0.00 ± 0.00
		E	-	0.06 ± 0.14	0.00 ± 0.00	0.12 ± 0.00	0.00 ± 0.00

* Treatments: meat analogs with different fat replacers, O (oil); EC (non-emulsified emulsion control); E (emulsion).

**Table 8:** TBARs (mg/kg) of meat analogs with different fat replacers (O, EC, and E) during the freezing storage (−18 °C, −60 °C) for six months.

TBARs (mg/kg)	**Storage** **Temperature (°C)**	Treatments *	**Storage (Months)**				
			**0**	**0.5**	**1**	**3**	**6**
	−18	O	0.18 ± 0.01^BC^	0.18 ± 0.02^BC^	0.18 ± 0.01^C^	0.19 ± 0.01^cB^	0.24 ± 0.01^bA^
		EC	0.18 ± 0.01^B^	0.18 ± 0.01^B^	0.18 ± 0.01^B^	0.39 ± 0.01^aA^	0.39 ± 0.02^aA^
		E	0.18 ± 0.01^C^	0.19 ± 0.02^C^	0.17 ± 0.01^C^	0.32 ± 0.03^bB^	0.43 ± 0.07^aA^
	−60	O	0.18 ± 0.01^BC^	0.17 ± 0.01^BC^	0.16 ± 0.01^bC^	0.19 ± 0.00^cB^	0.33 ± 0.04^cA^
		EC	0.18 ± 0.01^C^	0.17 ± 0.01^C^	0.18 ± 0.01^aC^	0.39 ± 0.01^aB^	0.50 ± 0.03^aA^
		E	0.18 ± 0.00^C^	0.17 ± 0.00^C^	0.17 ± 0.01^bC^	0.30 ± 0.01^bB^	0.45 ± 0.05^bA^

^a–c^ Means within a column with different letters indicate significant differences (*p* < 0.05). ^A–C^ Means within a row with different letters indicate significant differences (*p* < 0.05). * Treatments: meat analogs with different fat replacers, O (oil); EC (non-emulsified emulsion control); E (emulsion).

**Table 9:** Total viable counts and *Escherichia coli* count (log CFU/mL) of meat analogs with different fat replacers (O, EC, and E) during the freezing storage (−18 °C, and −60 °C) for six months.

	Storage Temperature (°C)	Treatments *	Storage (Months)				
			0	0.5	1	3	6
Total Viable Counts (log CFU/mL)	−18	O	1.26 ± 0.24^AB^	1.00 ± 0.00^B^	1.12 ± 0.16^bAB^	1.00 ± 0.00^B^	1.58 ± 0.35^aA^
		EC	1.28 ± 0.27	1.30 ± 0.00	1.35 ± 0.50^a^	1.15 ± 0.21	1.43 ± 0.13^ab^
		E	1.53 ± 0.14	1.15 ± 0.21	1.37 ± 0.29^b^	1.26 ± 0.24	1.15 ± 0.30^b^
	−60	O	1.26 ± 0.24^A^	1.10 ± 0.17^aA^	1.15 ± 0.21	1.00 ± 0.00^B^	1.31 ± 0.38^A^
		EC	1.28 ± 0.27^AB^	0.00 ± 0.00^bC^	1.00 ± 0.00	1.00 ± 0.00^B^	1.63 ± 0.13^A^
		E	1.53 ± 0.14^A^	1.00 ± 0.00^aB^	1.40 ± 0.3	1.00 ± 0.00^B^	1.37 ± 0.31^AB^
*Escherichia coli* (log CFU/mL)	−18	O	ND	ND	ND	ND	ND
		E	ND	ND	ND	ND	ND
		EC	ND	ND	ND	ND	ND
	−60	O	ND	ND	ND	ND	ND
		E	ND	ND	ND	ND	ND
		EC	ND	ND	ND	ND	ND

^a,b^ Means within a column with different letters indicate significant differences (*p* < 0.05). ^A,B^ Means within a row with different letters indicate significant differences (*p* < 0.05). * Treatments: meat analogs with different fat replacers, O (oil); EC (non-emulsified emulsion control); E (emulsion).

## Data Availability

Data is contained within the article.
